# Oncolytic viruses in lung cancer: mechanisms of action and research progress

**DOI:** 10.3389/fmicb.2026.1801703

**Published:** 2026-04-09

**Authors:** Tuo Ji, Xinyi Hu, Yuzhi Gao, Kun Yu, Jiachen He, Lin Wang, Xuzhu Gao

**Affiliations:** 1 Lianyungang Clinical College, Xuzhou Medical University, The Second People's Hospital of Lianyungang, Lianyungang, China; 2 Institute of Clinical Oncology, The Second People's Hospital of Lianyungang City (Cancer Hospital of Lianyungang), Lianyungang, China; 3 Department of Life Sciences, Bengbu Medical University, Bengbu, China

**Keywords:** anti-cancer mechanisms, combination therapy, immunotherapy, lung cancer, oncolytic viruses

## Abstract

Lung cancer remains the leading cause of cancer-related mortality worldwide, posing a profound public health challenge. Current primary therapeutic modalities-surgical resection, radiotherapy, and chemotherapy-often exhibit limited efficacy, accompanied by frequent adverse events and treatment resistance. Oncolytic viruses (OVs), an emerging class of anti-cancer therapeutics, can infect and lyse cancer cells while their effects on normal tissues are generally limited. Leveraging OVs in lung cancer therapy holds substantial promise for improving patient survival outcomes. This review comprehensively examines the multifaceted anti-cancer mechanisms of OVs, including induction of apoptosis, immunogenic cell death, and neoantigen presentation. Additionally, it explores promising combinatorial strategies, such as OV-based immunotherapy and targeted therapies. By synthesizing current evidence, this review aims to inform the optimization of OV-based therapeutic regimens for lung cancer, ultimately enhancing patient survival and quality of life while addressing limitations of conventional treatments.

## 1 Introduction

Lung cancer remains the leading cause of cancer-related mortality globally, with an estimated 2 million new cases and 1.76 million deaths annually (Thai et al., 2021). For early-stage lung cancer, surgical resection is the preferred treatment modality, whereas chemotherapy and radiotherapy (including neoadjuvant and adjuvant therapy) are standard for locally advanced and metastatic disease. However, these interventions often yield limited overall survival (OS) and are associated with significant toxicities (Duma et al., 2019). Biological therapy, despite its complexity, has emerged as the fourth pillar of cancer treatment due to its favorable efficacy profile, minimal side effects, and potential to alleviate patient suffering. In recent years, immunotherapy-by activating or enhancing the host immune system to target tumor cells-has revolutionized solid tumor treatment. Notably, anti-CTLA-4 monoclonal antibody therapy has demonstrated unprecedented long-term survival outcomes in metastatic melanoma patients. This has established immunotherapy as a new standard of care for selected lung cancer patients, with expectations for further expansion in the field (Christian et al., 2017).

Oncolytic viruses (OVs) represent a class of viruses that infect and lyse cancer cells while eliciting systemic antitumor immune responses. With the global and Chinese rise in malignant tumor incidence and the unmet need for effective therapies, OVs hold significant promise due to their multifaceted antitumor mechanisms: direct tumor cell lysis, immune system activation/modulation, and tumor microenvironment (TME) reprogramming. Combinatorial strategies pairing OVs with chemotherapy or immunotherapy have demonstrated potentiated therapeutic effects, offering new hope to cancer patients (Muthukutty and Yoo, 2023). Importantly, clinical trials to date have not reported treatment-related deaths or severe adverse events associated with OV therapy. As an emerging therapeutic modality, OVs exhibit tremendous potential to expand treatment options for cancer patients (Moumita et al., 2020).

## 2 Introduction to OVs

OVs are a class of viruses that infect and lyse cancer cells while eliciting systemic antitumor immune responses (Zhang et al., 2023). The earliest discovery of the oncolytic potential of viruses can be traced back to 1904, when tumor regression was observed in a female patient with leukemia following influenza infection. This suggested that viral infections might exert a potentially beneficial effect on tumors (Zheng et al., 2019). The historical trajectory of OV therapy began in 1912 when an Italian physician observed tumor regression in a cervical cancer patient following rabies vaccine administration (Das et al., 2022). This serendipitous observation spurred early investigations into using wild-type viruses for cancer treatment. From the 1950s to the 1970s, numerous clinical trials were conducted using wild-type viruses such as varicella-zoster and measles virus. However, constrained by limited medical resources, technical limitations, and nascent understanding of viral-tumor interactions, these pioneering efforts failed to revolutionize clinical practice, resulting in stagnated progress for decades (Asada, 1974). The field underwent a paradigm shift in 1991 with the first report of an oncolytic virus inhibiting glioma growth in murine models (Ezzeddine et al., 1991), marking the commencement of modern OV research. In 1996, the genetically modified adenovirus ONYX-015 was introduced into a phase I clinical trial for the treatment of head and neck cancers and other solid tumors. This was the first genetically engineered OV to enter human trials and represented a milestone in the field (Opyrchal et al., 2009). In the early 21st century, OV research entered a phase focused on enhancing therapeutic efficacy. Through methods such as random mutagenesis and insertion of immunomodulatory genes, researchers were able to significantly improve viral performance. For example, sensitivity to specific antibiotics was exploited to accelerate genomic engineering, facilitating the insertion of transgenes. Simultaneously, the identification of nonessential genes for viral replication in tumor cells allowed for rational optimization of safety and efficacy. Rezaei et al. (2025) combined the Sleeping Beauty transposon system with long-read nanopore sequencing to construct insertional mutant libraries of herpes simplex virus type 1 (HSV-1) and vaccinia virus, enabling the identification of stable transgene integration sites and beneficial gene deletions that enhanced viral performance. In another study, a “directed natural evolution” strategy was employed to generate a next-generation OV (NGOVM) in refractory colorectal cancer cells. This virus exhibited nearly 10,000-fold higher lytic potency compared with conventional OVs and acquired mutations that enhanced receptor binding and interferon resistance (Guo et al., 2023). In terms of clinical application, in 2005, the China National Medical Products Administration approved a recombinant type 5 adenovirus (H101), with deletions in the E1B-55kD and E3 regions, for use in combination with chemotherapy for nasopharyngeal carcinoma. This was the first approved OV product worldwide (Liang, 2018). A pivotal milestone occurred in 2015 when the FDA approved talimogene laherparepvec (T-VEC), an HSV-1 derived OV, which reinvigorated translational efforts for OV-based cancer therapies (Terrível et al., 2020). This pivotal development reignited interest in virotherapy. Since then, OV research has entered a rapid development phase, characterized by diversified platform technologies such as vaccinia virus, Newcastle disease virus (NDV), and vesicular stomatitis virus, the mainstream adoption of combination therapies, and the global expansion of research pipelines. In particular, the vaccinia virus platform has attracted significant attention due to its synergistic potential when combined with immune checkpoint inhibitors, CAR-T cells, and other immunotherapies (Jin et al., 2025). To date, more than 30 OVs have entered clinical trials. One of the most notable examples is JX-594, a vaccinia virus with inactivated thymidine kinase, engineered to carry granulocyte-macrophage colony-stimulating factor (GM-CSF) and lacZ transgenes. JX-594 exerts its antitumor effects through both replication-dependent oncolysis and the induction of immune responses. Clinical studies have shown that JX-594 is safe and consistently induces tumor necrosis in patients with various solid tumors (Parato et al., 2012). The 2017 clinical report demonstrating synergistic efficacy of T-VEC combined with the PD-1 inhibitor Keytruda in melanoma treatment (Ribas et al., 2017) further catalyzed interest in OV combination strategies (Table 1).

**Table 1:** Development of oncolytic viruses.

Development stage	Time	Development events	References
Discovery stage	1904–1990	In 1904, revealing viral infections may benefit tumors.	Zheng et al., 2019
		In 1912, the start of viral tumor therapy.	Das et al., 2022
Modification stage	1991–2000	In 1991, scientists first used oncolytic viruses to inhibit the growth of gliomas in mice.	Ezzeddine et al., 1991
		In 1996, the genetically modified adenovirus ONYX-015 entered Phase I clinical trials.	Opyrchal et al., 2009
Efficiency improvement stage	21st century	In 2005, the oncolytic virus H101 was approved for nasopharyngeal cancer treatment.	Liang, 2018
Rapid development stage	2015–present	In 2015, the FDA approved the oncolytic virus product T-Vec for market.	Terrível et al., 2020
		In 2017, T-VEC combined with PD-1 drug Keytruda for melanoma treatment.	Ribas et al., 2017

**Figure 1:**
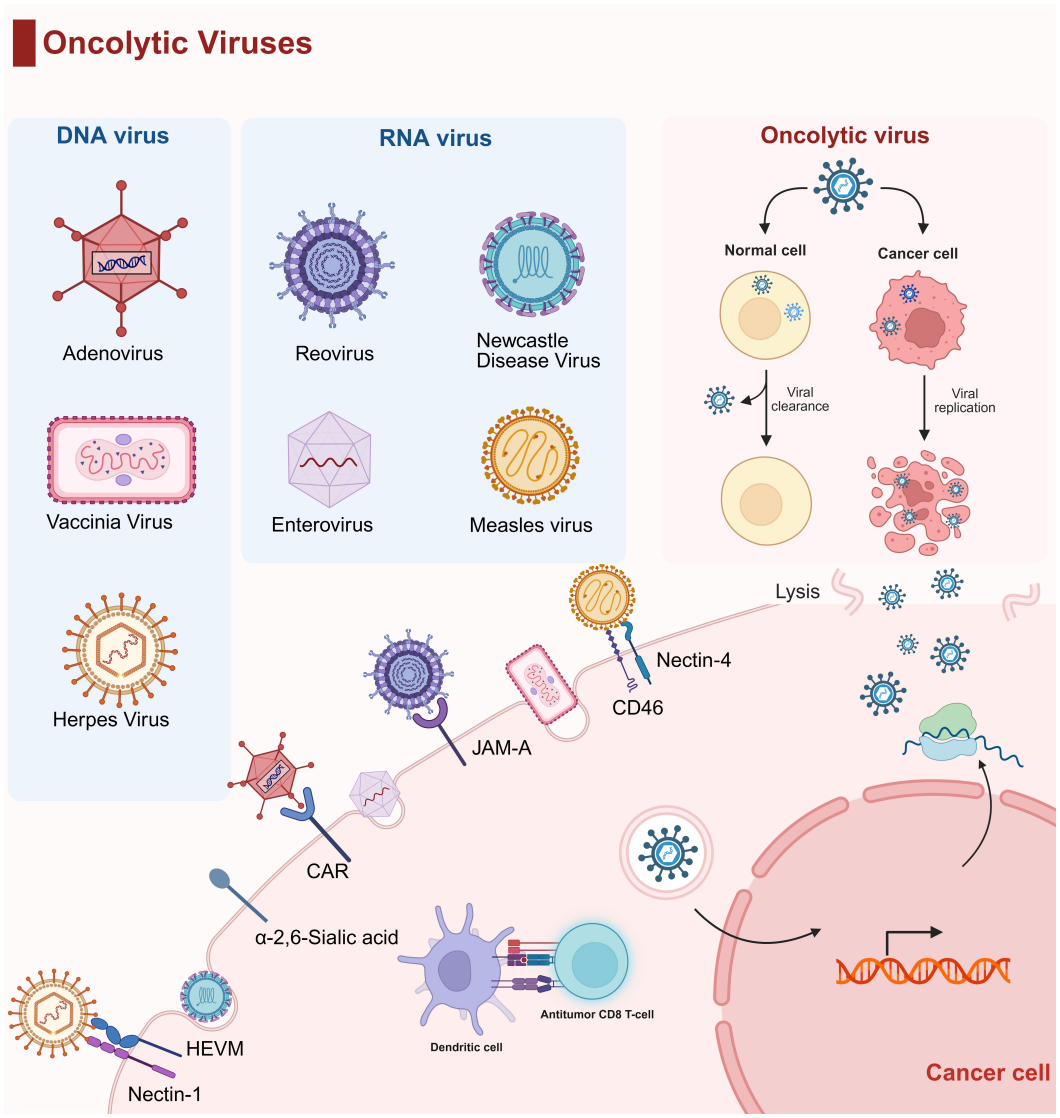
The features of oncolytic viruses. OVs are divided into DNA viruses and RNA viruses, each of which utilizes different cell surface receptors to enter cancer cells. In normal cells, virus replication is limited by innate immune clearance, while in cancer cells, defective antiviral pathways allow selective virus replication, leading to direct oncolysis and release of tumor-associated antigens. This process activates dendritic cells and antitumor CD8^+^ T cells, triggering systemic antitumor immunity.

OVs are currently classified into two primary categories: naturally occurring viruses and genetically engineered viruses. Naturally occurring OVs include adenoviruses, reoviruses, NDV, enteroviruses, and measles virus (MV), with commonly used types encompassing adenoviruses, reoviruses, NDV, enteroviruses, MV, vaccinia virus, and herpesviruses (Figure 1). In terms of advantages, these viruses generally possess tumor cell targeting or specific infectivity, capable of reducing the number and volume of tumor cells through cascading oncolytic processes (Singh et al., 2012). Most can serve as gene vectors for carrying therapeutic genes, with clear genome structures or sufficient research basis, facilitating genetic engineering modification (Thomas et al., 2003). Some can also activate the immune system to enhance antitumor effects; notably, vaccinia virus, with a long history as a vaccine vector, has well-defined safety and immune mechanisms (Jacobs et al., 2009). However, they share common disadvantages: as external pathogens, they induce immunogenicity; tumor cell heterogeneity tends to result in inconsistent infection and killing effects, often requiring multiple treatments or combination with other methods (McDonald et al., 2019). Additionally, each virus has unique issues: adenoviruses may cause toxicity in normal cells in some cases (Farrera-Sal et al., 2021; Zhao et al., 2021); reoviruses are limited by administration routes and susceptible to the impact of chemotherapy drugs (Lee et al., 2024); NDV carries potential neurotoxicity (Moura et al., 2016); enteroviruses are sensitive to environmental conditions (such as temperature and pH) and may exert side effects on normal tissues (Liew and Gerba, 1980); vaccinia virus face difficulties in targeting deep or multiple tumors during intratumoral injection and uneven virus distribution in intravenous injection (Breitbach et al., 2011); herpes viruses pose a risk of neurotoxicity due to their neurotropic nature (Smith, 2012) (Table 2). To enhance tumor cell tropism, selective replication capacity, and lytic potential, as well as to augment host antitumor immune responses, most OVs undergo genetic modification (Zhu et al., 2023). For example, the adenovirus Ad-TD-nsIL12, by deleting the E1A-CR2 gene, restricts viral replication to tumor cells, thereby enhancing its targeting specificity and reducing toxicity to normal cells. Deletion of the E1B-19K gene accelerates apoptosis in infected tumor cells, thereby enhancing its capacity to eliminate tumor cells. Furthermore, deletion of the E3-gp19K gene facilitates the intracellular presentation of tumor-specific antigens, thereby potentiating the antitumor efficacy (Ning et al., 2024). OVs have emerged as a novel cancer therapy strategy. Their replication tends to occur more efficiently in tumor cells, causing their lysis and death. This relative selectivity stems from the abnormal activation of certain signaling pathways in tumor cells, which creates a favorable environment for viral replication. OVs also release tumor antigens and pathogen-associated molecular patterns, promoting dendritic cell maturation and activation, and in turn, activating tumor-specific T cells. Moreover, they can alter the TME, shifting it from an immune-suppressive to an immune-activated state, thus enhancing immune cell attack on tumors (Bommareddy et al., 2018). OVs have shown potential in treating various cancers. For instance, M032 is a genetically engineered oncolytic HSV-1 that can express interleukin-12 (IL-12). Combined with surgery, it may prolong the survival period of dogs with spontaneous gliomas (Omar et al., 2021). The oncolytic adenovirus ORCA-010 can activate pro-inflammatory myeloid cells and, when combined with PD-1 blockade, promote T cell recruitment and activation, enhancing the immune response to tumors. By modulating immune cells in the TME, ORCA-010 can improve the efficacy of immune checkpoint inhibitors, offering a new strategy for melanoma treatment (Milenova et al., 2021). Additionally, OVs are being studied in other cancers, such as non-small cell lung cancer, head and neck cancer, glioblastoma, and hepatocellular carcinoma. These studies typically involve OVs used alone or in combination with other therapies like chemotherapy, radiotherapy, targeted therapy, and immunotherapy.

**Table 2:** Characteristics of commonly used oncolytic viruses.

Virus type	Advantages	Disadvantages	References
Adenovirus	1. High gene carrying capacity;	Hepatotoxicity	Thomas et al., 2003; Singh et al., 2012; McDonald et al., 2019; Farrera-Sal et al., 2021; Zhao et al., 2021
	2. Mature genome manipulation;		
	3. Wide clinical application.		
Reovirus	1. High safety;	1. Drug resistance;	Thomas et al., 2003; Singh et al., 2012; McDonald et al., 2019; Lee et al., 2024
	2. Ras signaling pathway dependence;	2. Chemotherapy drugs may affect activity.	
	3. Effective oral administration;		
	4. Trojan horse delivery via virus–antibody complex and monocytes.		
Newcastle disease virus	1. No neutralizing antibodies in humans, enabling repeated intravenous injection;	Potential neurotoxicity	Thomas et al., 2003; Singh et al., 2012; Moura et al., 2016; McDonald et al., 2019
	2. No specific receptor required;		
	3. Immune evasion via syncytium formation.		
Enterovirus	1. Diverse receptors;	Sensitivity to environmental conditions (temperature, pH), strict storage, and transportation conditions	Liew and Gerba, 1980; Thomas et al., 2003; Singh et al., 2012; McDonald et al., 2019
	2. Small and simple genome, easy for genetic modification;		
	3. Upregulates pro-apoptotic factor PMAIP1 to induce tumor cell apoptosis.		
Measles virus	1. High safety;	Sensitivity to chemotherapy drugs impacts its activity	Thomas et al., 2003; Singh et al., 2012; McDonald et al., 2019
	2. Induces glycolysis in glioma cells;		
	3. Well-understood biological properties.		
Vaccinia virus	1. Long history and high safety profile;	Uneven virus distribution in intravenous injection affecting efficacy	Jacobs et al., 2009; Breitbach et al., 2011; Singh et al., 2012; McDonald et al., 2019
	2. Large genome, allowing insertion of foreign genes;		
	3. Short replication cycle.		
Herpes virus	1. DNA virus, capable of stably inserting multiple foreign genes;	Potential neurotoxicity	Thomas et al., 2003; Singh et al., 2012; Smith, 2012; McDonald et al., 2019
	2. Kills cancer stem cells resistant to chemotherapy and radiotherapy.		

## 3 Mechanisms of OVs in lung cancer

Recent research has highlighted OVs as a promising new strategy for lung cancer treatment, with their mechanisms of action involving several key aspects.

### 3.1 Direct tumor cell lysis

OVs exert antitumor effects by exploiting tumor-specific abnormalities in signaling pathways, enabling selective replication within cancer cells and subsequent cell lysis. Among these, aberrant activation of the RAS/RAF/MEK/ERK signaling pathway is considered a key mechanism underlying the selective replication of OVs. For instance, studies have demonstrated that vesicular stomatitis virus replicates more efficiently in tumor cells with sustained activation of this pathway. Specifically, tumor cells overexpressing RAS or RAF1 exhibit increased sensitivity to VSV-mediated oncolysis while showing reduced responsiveness to type I interferon therapy. Treatment with MEK inhibitors can partially restore interferon sensitivity and suppress viral replication activity (Noser et al., 2007). Moreover, research on the oncolytic vaccinia virus JX-594 further supports this mechanism. JX-594 selectively replicates and lyses tumor cells through mechanisms dependent on the activation of the EGFR/RAS signaling pathway, as well as on elevated expression of intracellular tyrosine kinases and resistance to type I interferons (Parato et al., 2012). This is in contrast to normal cells, where antiviral mechanisms limit viral spread and prevent apoptosis or necrosis, thus inhibiting OV replication. For example, in epithelial-rich tissues such as the lungs, ASK2-dependent apoptosis may contribute to antiviral defense by eliminating infected cells in tissues characterized by rapid turnover and regenerative capacity (Okazaki et al., 2015). In lung cancer cells, the replication of OVs leads to the accumulation of double-stranded RNA, activating the antiviral protein kinase PKR This activation inhibits protein synthesis in the tumor cells, ultimately triggering apoptosis (Errington et al., 2008). For example, Coxsackievirus B5 (CV-B5) can target specific receptors on the surface of tumor cells, selectively infecting and killing them through apoptosis and autophagy induction without harming normal cells. This selective mechanism makes CV-B5 a potential treatment for refractory non-small cell lung cancers (NSCLC) (Cui et al., 2023). LHPP has been identified as a novel tumor suppressor, and GD55-LHPP, a recombinant oncolytic adenovirus engineered to express LHPP, has been shown to effectively inhibit proliferation of lung cancer cell lines while exerting minimal effects on normal cells. Mechanistically, GD55-LHPP suppresses tumor growth in murine lung cancer xenograft models by activating apoptosis- and autophagy-related signaling pathways, thereby prolonging survival (Zhao et al., 2024). Apoptin, a low molecular weight apoptosis-inducing protein derived from the chicken anemia virus, selectively kills a variety of human cancer cells without harming normal cells (Noteborn, 2009; Zhu et al., 2021). Song et al. developed a recombinant adenoviral vector, Ad-Apoptin, based on human adenovirus type 5 to express apoptin in tumor cells. The expressed apoptin targets PKM2, suppressing glycolysis and cell proliferation in A549 cells, while promoting autophagy and apoptosis via the PKM2/AMPK/mTOR signaling axis (Song et al., 2024). Coxsackievirus A11 (CVA11) exhibits broad oncolytic activity across multiple NSCLC cell lines, with higher cytotoxicity observed in cells expressing elevated levels of ICAM-1. Mechanistic studies revealed that CVA11-induced cell death is partially apoptosis-dependent, as indicated by PARP cleavage, increased calreticulin expression, and HMGB1 release, suggestive of immunogenic cell death. *In vivo*, intratumoral injection of CVA11 led to complete regression of NSCLC xenografts without significant toxicity, highlighting its therapeutic potential and safety profile (Sakamoto et al., 2023).

### 3.2 Immune system activation

Under normal conditions, the immune system plays a crucial role in monitoring and eliminating abnormal cells in the human body. However, cancer cells often evade immune surveillance by downregulating major histocompatibility complex molecules, reducing antigen presentation, or secreting immunosuppressive factors such as TGF-β, IL-10, and PD-L1 (Binnewies et al., 2018). OVs can activate the host's immune system. They release tumor antigens, promoting dendritic cell maturation and T cell activation, thus triggering a specific immune response against the tumor (Mealiea and McCart, 2022). This immune activation helps eliminate residual tumor cells and provides long-term immune surveillance to prevent recurrence. For example, enadenotucirev (formerly known as colad1), a tumor-selective chimeric adenovirus, induces a local strong antitumor immune response in resectable NSCLC, such as increased CD8^+^ cell infiltration (Garcia-Carbonero et al., 2017). In lung cancer mouse models, vaccines with oncolytic adenovirus or vaccinia virus-infected reprogrammed tumor cells elicit tumor-specific T cell responses, significantly prolonging survival (Zhang et al., 2020). VACV GM-CSF^+^ is a genetically modified oncolytic vaccinia virus (VACV). It expresses granulocyte-macrophage colony-stimulating factor (GM-CSF), an immune-activating cytokine. GM-CSF promotes dendritic cell maturation and enhances antigen presentation. As a result, it can trigger T cell-mediated cytotoxic responses (Dougan et al., 2019). Patient-derived organoid models of NSCLC were used to evaluate the effects of these viruses. Researchers measured virus-induced tumor lysis and immune cell responses. VACV GM-CSF^+^ showed stronger immune activity than conventional VACV. In particular, organoids from patient 23T37 had increased levels of immune-related proteins. These included NCR1, KIR3DL1, CD27, CRTAM, FASLG, IFN-γ, and CXCL13, which are linked to NK and T cell activation (Lê et al., 2024).

### 3.3 Modulation of the TME

**Figure 2:**
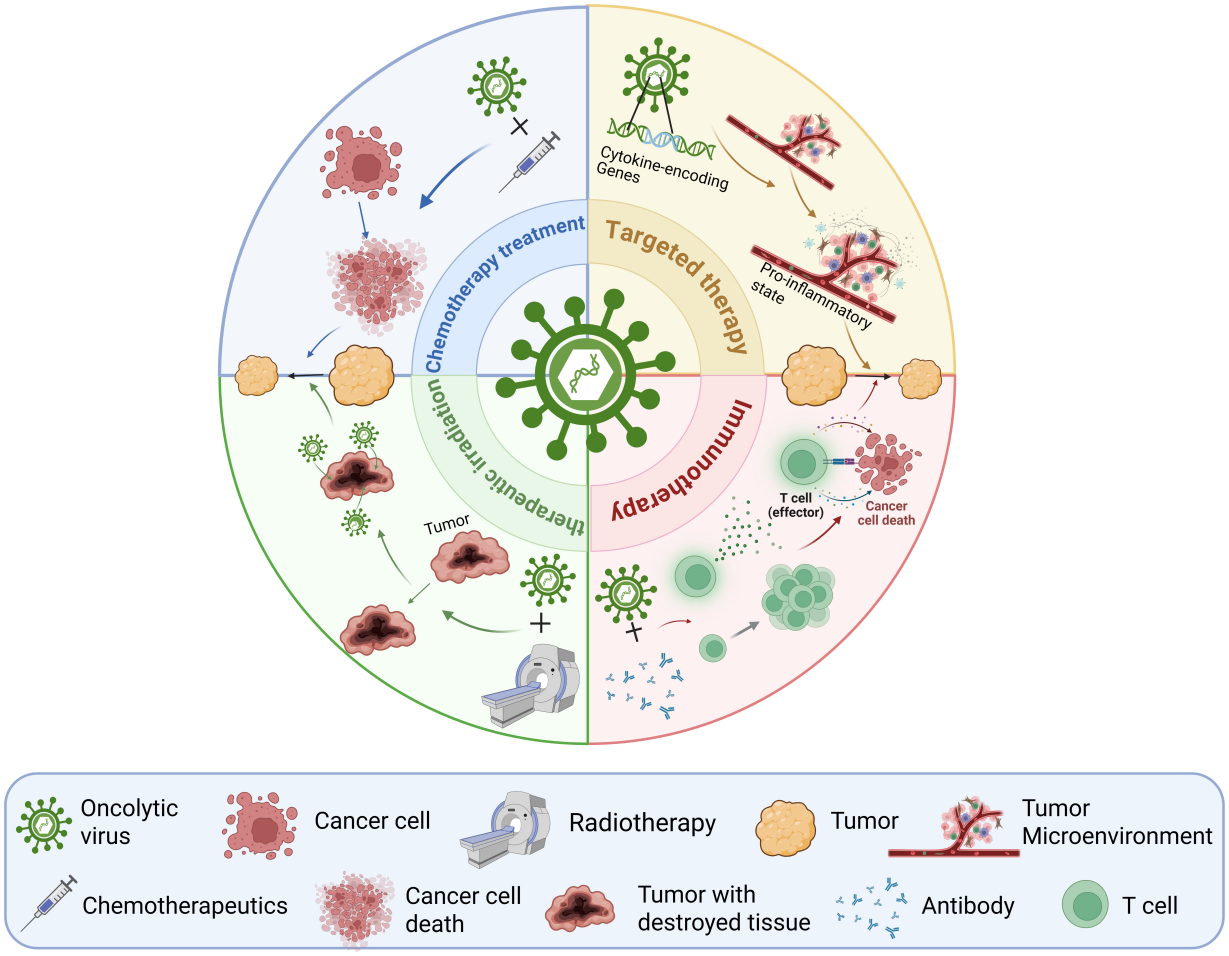
Combination therapy with oncolytic viruses. Through the combination of oncolytic viruses with various treatment methods, the anti-cancer effect is enhanced in multiple dimensions, including increasing the sensitivity of tumor cells to chemotherapy, altering the tumor microenvironment to cooperate with targeted therapy, synergistically enhancing the efficacy with radiotherapy, and activating immune responses to amplify the effect of immunotherapy. When chemotherapeutic drugs are combined with oncolytic viruses, the toxicity of chemotherapeutic drugs can be enhanced, and the sensitivity of tumor cells to viruses is increased, thereby achieving a more significant tumor regression effect; when radiotherapy is combined with oncolytic viruses, radiation can disrupt the tumor tissue structure, promote the entry of oncolytic viruses into tumor cells, and thus strengthen the antitumor immune effect of oncolytic viruses; when antibodies are combined with oncolytic viruses, oncolytic viruses can enhance the activation of tumor-specific lymphocytes and the proliferation of tumor antigen-specific CD8^+^ T cells, thereby killing tumor cells; when targeted therapy is combined with oncolytic viruses, oncolytic viruses encoding cytokines can reshape the tumor microenvironment into a pro-inflammatory state, leading to a more remarkable tumor regression effect.

The TME is the environment surrounding a tumor, composed of various cell types, including immune cells, extracellular matrix, blood vessels, and cancer-associated fibroblasts (Xiao and Yu, 2021). The interaction between the TME and cancer cells significantly influences numerous cellular processes related to tumor growth, invasion, and metastasis (Hofmann et al., 2014). The TME often exhibits immune-suppressive properties; tumors secrete chemokines and cytokines that inhibit the maturation and activation of dendritic cells, including the recognition and presentation of tumor antigens by antigen-presenting cells (Xiao and Yu, 2021). Moreover, tumor cells can promote the generation and activation of immune-suppressive cell populations, such as regulatory T cells, myeloid-derived suppressor cells, tumor-associated macrophages, and tumor-associated neutrophils (Guo and Wang, 2025). Upon infecting tumor cells, OVs induce the production of various cytokines and chemokines. These factors attract more immune cells, such as T cells and natural killer cells, into the TME while reducing immune-suppressive cells and molecules, thereby enhancing the antitumor immune response (Figure 2) (Nguyen et al., 2022; Harrington et al., 2019). For instance, the fusogenic oncolytic vaccinia virus improves the tumor immune microenvironment, exerting a more effective cytopathic effect and inducing immunogenic cell death in human and mouse lung cancer cells (Nakatake et al., 2021). Another study confirmed that OVs can infect tumor-infiltrating Tregs, leading to virus-mediated Treg depletion and subsequent tumor regression (Kristin et al., 2020). TG6050 is a novel VACV engineered to express both IL-12 and an anti-CTLA-4 antibody. It combines three key elements: an OV backbone, IL-12, and anti-CTLA-4. This design enables synergistic effects. In the LLC1 mouse lung cancer model, TG6050 induced marked tumor regression. More importantly, it triggered a strong adaptive immune response and reshaped the TME to further enhance antitumor activity (Azar et al., 2024). Ye et al. developed a double-deleted vaccinia virus expressing murine IL-9 (vvDD-IL-9). Its antitumor effects were tested in colorectal and lung cancer models. This virus significantly upregulated Th1-type chemokines and antitumor effectors such as IFN-γ, granzyme B, and perforin. It increased the percentages of CD4^+^ and CD8^+^ T cells, as well as Tregs, in the TME. At the same time, it reduced the proportion of immunosuppressive MDSCs induced by conventional OVs. These changes led to enhanced antitumor effects. Moreover, vvDD-IL-9 treatment upregulated immune checkpoint molecules including PD-1, PD-L1, and CTLA-4, and effectively slowed tumor growth (Ye et al., 2024).

### 3.4 Genetic engineering enhancement

Some OVs are genetically engineered to enhance their tumor selectivity and immune-activation capabilities, with the core design principles focusing on achieving tumor cell-specific replication and targeted infection of cancer cells. The construction of tumor-selective oncolytic viruses primarily relies on two technical approaches. One is the use of cancer-specific promoters to tightly control the expression of viral replication-related genes, thereby restricting viral replication to tumor cells with specific molecular characteristics and completely abrogating viral proliferation in normal cells. The other is the development of chimeric viruses via engineering the viral envelope, which endows the virus with the ability to specifically recognize and infect tumor cells by targeting tumor-associated surface receptors, while avoiding the binding and infection of normal tissue cells.

For the cancer-specific promoter-based strategy, the oncolytic adenovirus DNX-2401 is modified with the insertion of an RGD-4C motif, which boosts its ability to infect tumor cells while reducing normal cell infection (Gállego Pérez-Larraya et al., 2022). In the adenovirus platform, Chen et al. developed an engineered oncolytic adenovirus named Ad.TJL. It utilizes the human c-Met promoter to enhance tumor specificity and safety, incorporates a CMV-driven luciferase reporter, and retains the E1B-55K deletion found in Onyx-015. Ad.TJL selectively replicates in c-Met-high-expressing lung cancer cells, induces prominent cytopathic effects in multiple cell lines (e.g., A549, PC14PE6, CL1-5), and significantly suppresses tumor growth in mouse models, demonstrating robust oncolytic activity and targeting capacity (Chen S. Y. et al., 2024). Meissner et al. engineered a replication-deficient low pathogenic influenza A virus lacking the HA gene (ΔHA IAV). By modifying the viral envelope with a recombinant HA protein, they achieved single-round infection. Furthermore, the introduction of the murine IFN-γ gene yielded ΔHA-mIFN-γ, which, when administered intratracheally in transgenic NSCLC mice, led to tumor destruction and robust activation of NK cells, peripheral macrophages, and cytotoxic T cells. Notably, the virus polarized tumor-associated alveolar macrophages toward a pro-inflammatory M1 phenotype, thereby enhancing antitumor immunity (Meissner et al., 2023). In the chimeric virus platform, enadenotucirev is constructed through genetic engineering by precisely inserting the E2B gene region of type 3 adenovirus into the backbone of type 11p adenovirus, and deleting key fragments in the E3 and E4 regions (Fakih et al., 2023), and its clinical potential has been further validated in early-phase trials (Moreno et al., 2021). OncoViron is a chimeric broad-spectrum oncolytic adenovirus featuring synergistic and synergistic immunotherapy with multiple mechanisms. It possesses a triple tumor-targeting regulatory mechanism, three viral structural gene modifications, chimerization of three serotypes of adenoviruses, and loading of three types of anti-cancer immune genes. It effectively reshapes the tumor microenvironment to enhance the efficacy of immunotherapies such as PD-1 antibody and CAR-T cell therapy (Su et al., 2022). In the VACV platform, Nguyen et al. developed a triple gene-deleted oncolytic VACV (3KO RT) lacking TK, A46R, and VGF. It demonstrated potent oncolytic activity and favorable safety in multiple human cancer cell lines. By fusing the human CD55 domain to the A33R envelope protein, the virus showed improved resistance to complement-mediated clearance. Animal studies confirmed its ability to selectively target tumors via intravenous injection, expand *in vivo*, and maintain stable therapeutic payload expression in immunocompetent mouse models (Nguyen et al., 2025). Two novel Seneca Valley Virus (SVV) mutants, SVV-S177A and SVV-S177A/P60S, designed through structural biology and reverse genetics, exhibited improved infectivity or reduced immunogenicity. In athymic NSCLC mouse models, both mutants significantly extended median overall survival (Zhao et al., 2023). Advances have also been made in other OV platforms. The measles virus has been genetically modified into an aerosolized OV designed to target EGFR-mutant NSCLC. Through precise engineering of its envelope proteins, the virus is equipped with tumor-specific affinity ligands that enable high-efficiency infection and selective recognition of EGFR-expressing tumor cells within the lung tissue. This aerosol delivery approach holds significant potential to enhance local pulmonary administration while reducing systemic toxicity (Choi and Mesiano, 2025). In addition, a recombinant cowpox virus expressing tumor necrosis factor-related apoptosis-inducing ligand (TRAIL), known as vTRAIL, significantly inhibited tumor growth in lung cancer mouse models, demonstrating strong tumor selectivity and immune activation (Yoo, 2023). Similarly, the type 2 oncolytic herpes simplex virus (HSV-2), OH2, was engineered to express the VP5 protein, which enhanced its oncolytic activity in A549 cells and promoted apoptosis both *in vitro* and *in vivo* (Wang et al., 2024). Gao et al. introduced the CLEC2A gene into a vaccinia virus (VV) backbone, generating a replication-competent therapeutic virus named oncoVV-CLEC2A. This construct improved viral replication and effectively suppressed proliferation of the H460 lung cancer cell line (Gao et al., 2024). Beyond single-virus platforms, cell-based delivery systems have also attracted increasing attention. ALO/CELYVIR consists of bone marrow-derived mesenchymal stem cells (BM-MSCs) carrying the oncolytic adenovirus ICOVIR-5 (Cascallo et al., 2007). Studies have shown that menstrual blood-derived mesenchymal stem cells (MenSCs), when cultured under hypoxic conditions, display enhanced tumor-homing ability. When used as carriers for ALO/CELYVIR, these cells efficiently delivered the virus to subcutaneous A549 lung adenocarcinoma xenografts in mice, enabling localized viral release and promoting immune-mediated tumor clearance by the host (Costa-Garcia et al., 2024).

In summary, multiple genetically engineered OV platforms are rapidly advancing toward clinical application in lung cancer. These approaches show excellent efficacy and safety, particularly in the context of combination immunotherapy and targeted delivery. In addition to their direct oncolytic effects, engineered OVs can remodel the tumor immune microenvironment, enhance the response to radiotherapy and immune checkpoint inhibitors, and offer novel opportunities for combinatorial treatment strategies. Genetic engineering strategies include modulating chemokine expression, incorporating immune checkpoint targets, and enhancing immunogenic cell death, highlighting the broad therapeutic potential of OVs in lung cancer treatment.

OVs show broad prospects in lung cancer treatment. However, challenges remain in ensuring effective delivery, enhancing tumor-tissue targeting and infectivity, and managing potential immune-mediated side effects. Future research will delve deeper into these mechanisms, aiming to refine the design and application of OVs to boost treatment efficacy.

## 4 Combination therapy strategies with OVs

Combination therapy using OVs for lung cancer is an innovative strategy that combines the direct tumor-cell killing of OVs with the systemic advantages of other treatments. While this approach may increase local toxicity, it enhances treatment efficacy (Liu et al., 2022).

### 4.1 Combination of OVs and chemotherapy

Chemotherapy is used pre-operatively (neoadjuvant) or post-operatively (adjuvant), and also for advanced lung cancer. However, it offers low survival rates and high recurrence risks. When combined with OVs, chemotherapy's cytotoxic effects are enhanced, and tumor cells may become more susceptible to the viruses, leading to better tumor regression (Zhang and Cheng, 2020). For instance, the combination of oncolytic adenovirus and temozolomide induces apoptosis, viral replication, and autophagy, promoting lung cancer cell death (Gomez-Gutierrez et al., 2016). TG4010 is a recombinant viral vector based on the modified vaccinia Ankara (MVA) strain, a highly attenuated vaccine virus. It expresses both MUC1, a tumor-associated antigen, and IL-2. A multicenter, randomized phase II clinical trial demonstrated that TG4010 combined with standard chemotherapy can treat advanced NSCLC (Ramlau et al., 2008). Additionally, this combination therapy may allow for lower chemotherapy doses, reducing side effects. In addition, a study showed that a genetically modified oncolytic myxoma virus (MYXV) displayed potent tumor-specific cytotoxicity in both classical and variant subtypes of small cell lung cancer (SCLC), including cisplatin-resistant models. The combination of MYXV with low-dose cisplatin was found to be effective (Kellish et al., 2019). Also, a clinical study demonstrated that the type 3 Dearing strain reovirus (Reolysin), when combined with paclitaxel and carboplatin, was well tolerated and improved clinical outcomes in NSCLC patients (Villalona-Calero et al., 2016). This provides new hope for patients who are resistant to conventional therapies.

### 4.2 Combination of OVs and radiotherapy

For patients who are not candidates for surgery, radiotherapy is often the preferred treatment option. Radiotherapy has shown significant immune-editing activity in lung cancer (Taunk et al., 2017), which suggests that combining it with OVs could be synergistically effective. Combined radiotherapy can disrupt tumor tissue structure, enhancing oncolytic virus entry into tumor cells. Meanwhile, OVs can boost the antitumor immune effects of radiotherapy, improve local control and reduce the risk of tumor recurrence and metastasis. For example, the oncolytic herpes simplex virus (HSV) G207 combined with radiotherapy has been used to treat recurrent malignant gliomas (Markert et al., 2014). In the context of lung cancer, Li et al. developed a novel HSV-1 amplicon virus named AP27i145, in which four copies of the miRNA-145 target sequence were inserted into the 3′ untranslated region (UTR) of the essential viral gene ICP27. When combined with radiotherapy, AP27i145 selectively killed NSCLC cells while minimizing off-target effects on normal tissues (Li et al., 2013). In another study, Liu et al. engineered a recombinant NDV (NDV-anti-VEGFR2) that targets vascular endothelial growth factor receptor 2 (VEGFR2). Combined with radiotherapy, NDV-anti-VEGFR2 downregulated HIF-1α expression, alleviated tumor hypoxia, and interfered with DNA repair pathways, thereby significantly inhibiting tumor growth *in vitro* and *in vivo* while increasing radiosensitivity. The research team also established a large-scale NDV production and purification process using mammalian cells, laying the foundation for future clinical translation (Liu et al., 2025). These examples illustrate that the combination of OV therapy and radiotherapy for lung cancer has already shown certain potential and promise.

### 4.3 Combination of OVs and immunotherapy

Immunotherapy can enhance antitumor immune responses, counteract tumor-induced immune suppression, and boost immune system efficacy. OVs induce tumor cell death, further activating and infiltrating immune cells to enhance tumor recognition and attack. Immunotherapy sustains and amplifies the OV-triggered antitumor immune response. Studies show that the combination of a Delta-24-RGDOX oncolytic adenovirus (engineered to express the target protein) and anti-PD-L1 antibodies significantly enhances the activation of tumor-specific lymphocytes and the proliferation of tumor-antigen-specific CD8^+^ T cells, thereby inducing cancer-specific immunity (Jiang et al., 2017). This combination therapy can improve lung cancer treatment, control tumor progression, and potentially extend survival and enhance quality of life. For instance, T-VEC combined with the PD-1 antibody pembrolizumab boosts objective response rates in advanced melanoma patients (Chesney et al., 2023b). Oncolytic vaccinia virus-based triple therapy, blocking PD-1 or TIM-3 induced T-cell exhaustion, is more effective for refractory lung cancer (Sun et al., 2020). In a murine NSCLC model, co-administration of anti-PD-1 antibody with a recombinant adenovirus encoding TNFα and IL-2 (Ad5-CMV-mTNFα/mIL-2) significantly increased the proportion of cytotoxic tumor-infiltrating lymphocytes (TILs) and inhibited tumor growth (Kudling et al., 2023). In addition, VVL-m12, a genetically modified oncolytic vaccinia virus developed by Chen et al., was engineered to express IL-12 and exhibited enhanced replication and cytotoxicity. Compared to conventional vaccinia virus (VV), VVL-m12 demonstrated superior antitumor efficacy in subcutaneous lung cancer models. Mechanistically, it promoted infiltration of CD4^+^ and CD8^+^ T cells, activated dendritic cells, induced M2-to-M1 macrophage polarization, and inhibited tumor angiogenesis. More importantly, VVL-m12 enhanced the tumor's responsiveness to α-PD-1 therapy, indicating its potential utility in combination immunotherapy for lung cancer (Chen L. et al., 2024). In clinical investigation, a phase II trial for metastatic NSCLC (the STOMP study) demonstrated that adenoviral delivery of herpes simplex virus thymidine kinase (ADV/HSV-tk), combined with valacyclovir gene therapy and stereotactic body radiotherapy (SBRT) prior to pembrolizumab administration, could enhance the antitumor effect of pembrolizumab (Guan et al., 2024). At the same time, oncolytic NDV (ONDV)-derived therapies have shown good safety profiles and antitumor activity in clinical studies, but as monotherapies, they are insufficient to cure solid tumors. Research has shown that in NSCLC models, ONDVs expressing IL-12 can enhance the antitumor efficacy and persistence of HER2-targeted CAR-T cells. The combination therapy was significantly more effective *in vivo* than either treatment alone, indicating that ONDVs may serve as effective enhancers of CAR-T cell therapy (Rosewell Shaw et al., 2024). Notably, the previously mentioned TG6050 also demonstrated enhanced antitumor activity when combined with anti-PD-1 antibody in the LLC1 murine lung cancer model (Azar et al., 2024). In summary, current findings strongly suggest that combination strategies involving OVs and immunotherapy can produce synergistic antitumor effects in various cancers, particularly in lung cancer. As research advances and technology evolves, the combination of OVs and immunotherapy holds promise as a key lung cancer treatment.

### 4.4 Combination of OVs and targeted therapy

**Figure 3:**
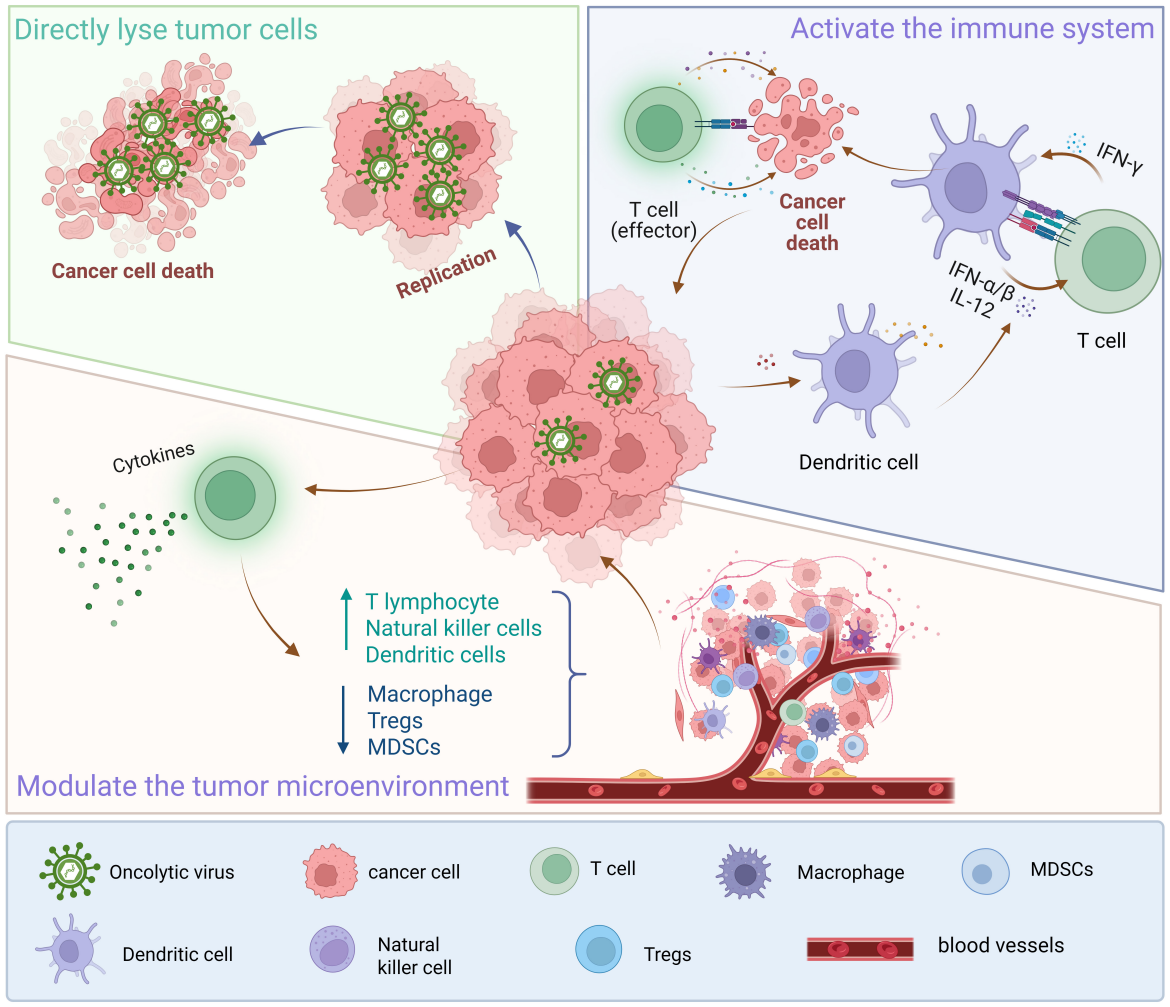
Mechanisms of action of oncolytic viruses. Oncolytic viruses kill tumor cells through multiple mechanisms, including directly lysing tumor cells, activating the immune system, and regulating the immune microenvironment. Oncolytic viruses can enter tumor cells and replicate effectively, thereby killing the tumor cells; meanwhile, after entering tumor cells, oncolytic viruses can induce dendritic cells to release tumor antigens, promote the maturation of dendritic cells and the activation of T cells, and thus trigger tumor-specific immune responses to eliminate tumor cells; furthermore, when oncolytic viruses infect tumor cells, they induce the production of various cytokines and chemokines. These factors recruit more immune cells into the tumor microenvironment, while reducing immunosuppressive cells and molecules, thereby enhancing the antitumor immune response and inducing tumor cell lysis.

Targeted therapies precisely hit specific molecular targets to curb cancer cell growth and spread. However, drug resistance may develop during treatment, reducing efficacy. OVs encoding cytokines can remodel the TME to a pro-inflammatory state and markedly upregulate PD-L1 expression, enhancing targeted therapy efficacy (Belcaid et al., 2020). For instance, combining an IL-7-encoding oncolytic adenovirus (oAD-IL7) with B7H3-targeted CAR-T cells shows synergistic antitumor activity in glioma treatment (Huang et al., 2021). In advanced melanoma patients, T-VEC combined with ipilimumab exhibits greater antitumor activity without extra toxicity than ipilimumab alone (Chesney et al., 2023a). In lung cancer, the combination of OVs and targeted therapies also shows promising potential. A case report described a SCLC patient who received intratumoral injection of recombinant human adenovirus type 5 along with targeted therapy, resulting in prolonged survival and a shift in immune status toward an inflammatory TME (Zhao et al., 2022). Moreover, Chen et al. developed Ad.TJL, an engineered oncolytic adenovirus, which exhibited significantly enhanced antitumor activity when combined with rapamycin, a selective mTOR inhibitor, in both *in vitro* and *in vivo* models. This synergy highlights the potential of Ad.TJL and rapamycin as a more effective combination strategy for cancer treatment (Chen S. Y. et al., 2024). Further research revealed that Coxsackievirus B5/Faulkner (CV-B5/F) exerts potential oncolytic effects in NSCLC by inducing apoptosis and autophagy. In refractory NSCLC cells, combining CV-B5/F with either a DNA-dependent protein kinase (DNA-PK) inhibitor or an ataxia-telangiectasia mutated (ATM) inhibitor produced synergistic effects, significantly enhancing cell death (Cui et al., 2023). Thus, combining OVs with targeted therapy could offer a new lung cancer treatment strategy (Figure 3).

## 5 Clinical application and development of OVs

As an emerging cancer therapy, OVs show broad prospects in clinical application (Macedo et al., 2020; Du et al., 2025). So far, four OVs have been approved for cancer treatment (Table 3). Beyond these, clinical trials for OVs are actively conducted for various solid tumors, including lung cancer and glioma, with promising initial results. Most OVs in current clinical trials are attenuated derivatives of common human pathogens. In recent years, genetic engineering has been applied to further reduce their pathogenicity, enhance their oncolytic ability, or increase their specificity to cancer tissues. For instance, measles virus is first attenuated through continuous passage in cell cultures and then genetically engineered to boost its oncolytic potency and tumor specificity (Kelly and Russell, 2007). Mechanistic studies using murine tumor models have shown that adenovirus-mediated delivery of membrane-stable CD40 ligand (MEM40) and interferon-β (IFNβ) can locally activate conventional dendritic cells, significantly enhance their function, and promote their migration to draining lymph nodes (Zheng et al., 2023). This activation induces a robust systemic CD8^+^ T cell response, leading to tumor regression through a cDC1-dependent mechanism. When combined with immune checkpoint inhibitors, this strategy can effectively control distant tumors and pulmonary metastases. Notably, in a phase I clinical trial involving patients with NSCLC (NCT05076760), the oncolytic adenovirus MEM-288, engineered to express both MEM40 and IFNβ, was shown to induce tumor cell loss, increase T cell infiltration, and expand tumor-specific T cell clones, demonstrating strong immunostimulatory potential (Zheng et al., 2023). Novel research and clinical achievements have further expanded the clinical application spectrum of oncolytic viruses in solid tumors. A recombinant Newcastle disease virus expressing porcine α1,3GT gene (NDV-GT) has been successfully developed, which exhibits excellent safety and antitumor efficacy via intravenous injection by triggering hyperacute rejection, providing an innovative technical strategy for tumor immunovirotherapy (Zhong et al., 2025). A multicenter phase I clinical trial confirmed that VG161, an oncolytic herpes simplex virus engineered to express IL-12, IL-15/IL-15Rα and a PD-1-PD-L1 blocking fusion protein, has favorable safety and significant antitumor efficacy in advanced hepatocellular carcinoma patients, and it can remodel the tumor immune microenvironment and restore sensitivity of drug-resistant tumors (Shen et al., 2025). In addition, a phase I clinical trial validated the good safety and preliminary antitumor efficacy of oncolytic adenovirus TILT-123 combined with pembrolizumab in platinum-resistant or refractory ovarian cancer patients, laying a foundation for the application of this combination regimen in ovarian cancer (Block et al., 2025). Taken together, as summarized in Table 4, an increasing number of OVs are undergoing clinical evaluation for lung cancer, with ongoing advances and new breakthroughs. These developments not only reinforce the clinical utility of OVs in cancer treatment, but also offer promising directions for the development of personalized combination strategies in future therapeutic approaches.

**Table 3:** Oncolytic viruses currently approved for marketing.

Oncolytic virus	Approval time	Indication	Country of approval	References
Rigvir	2004	For melanoma treatment	Latvia	Alberts et al., 2018
H101	2005	For advanced nasopharyngeal cancer treatment in combination with chemotherapy	China	Liang, 2018
T-VEC	2015	For inoperable advanced melanoma patients	USA and Europe	Andtbacka et al., 2015
G47Δ	2021	For malignant glioma treatment	Japan	Todo et al., 2022

**Table 4:** Clinical Trials of Lung Cancer-Associated Oncolytic Viruses.

Title	Registration number	Virus	Phase
Safety and efficacy of recombinant oncolytic adenovirus L-IFN injection in relapsed/refractory solid tumors clinical study	NCT05180851	YSCH-01	I
MEM-288 oncolytic virus alone and in combination with standard of care therapy in advanced solid tumors	NCT05076760	MEM-288	I
Binary oncolytic adenovirus in combination with HER2-specific autologous CAR VST, advanced HER2 positive solid tumors	NCT03740256	CAdVEC	I
SBRT and oncolytic virus therapy before pembrolizumab for metastatic TNBC and NSCLC	NCT03004183	ADV/HSV-tk	II
Oncolytic MG1-MAGEA3 with Ad-MAGEA3 vaccine in combination with pembrolizumab for non-small cell lung cancer patients (Pol et al., 2019; Jenner et al., 2021)	NCT02879760	Ad/MG1-MAGEA3	I/II
Mechanism of action trial of ColoAd1 (Garcia-Carbonero et al., 2017)	NCT02053220	Colo-Ad1	I
A clinical study on oncolytic virus injection (R130) for the treatment of relapsed/refractory advanced solid tumors	NCT05886075 NCT05961111 NCT05860374	R130	I
A Study of T3011 administered via intravenously in patients with advanced solid tumors.	NCT05598268	T3011	I/II
A clinical trial assessing BT-001 alone and in combination with pembrolizumab in metastatic or advanced solid tumors	NCT04725331	BT-001	I/II
Safety study of recombinant vaccinia virus to treat refractory solid tumors	NCT00625456	JX-594(Pexa-Vec)	I
CVA21 and pembrolizumab in NSCLC & bladder cancer (VLA-009 STORM/KEYNOTE-200) (Rudin et al., 2023)	NCT02043665	V937	I
Phase 2 study of REOLYSIN^®^ in combination with paclitaxel and carboplatin for non-small cell lung cancer with KRAS or EGFR activation	NCT00861627	REOLYSIN^®^	II
A study of oncolytic virus injection (RT-01) in patients with extensive-stage small cell lung cancer	NCT05205421	RT-01	I
Study of TBio-6517 given alone or in combination with pembrolizumab in solid tumors	NCT04301011	TBio-6517	I/II
Intrapleural administration of HSV1716 to treat patients with malignant pleural mesothelioma	NCT01721018	HSV1716	I/II
Intrapleural measles virus therapy in patients with malignant pleural mesothelioma	NCT01503177	MV-NIS	I

Data were collected from National Clinical Trials (http://clinicaltrials.gov/).

Despite their potential, OVs also have certain drawbacks that need to be overcome. Future research should focus on optimizing the genetic design of OVs, improving their targeting and killing ability for lung cancer cells, enhancing the safety of OV therapy, and developing more clinically applicable OVs. Additionally, many questions about combination therapies of OVs with other drugs, such as their efficacy, safety, administration order, time interval, and optimal delivery route, remain to be answered. Besides continuing to develop more effective OV drugs, it is important to create combination therapies that can synergize with existing lung cancer treatments to improve treatment plans and patient outcomes.

## 6 Advantages and disadvantages of OVs therapy for lung cancer

Compared to other malignancies, OVs therapy offers several distinct advantages in the treatment of lung cancer. First, the unique anatomical characteristics of the lungs provide a feasible route for aerosolized inhalation delivery (Liang et al., 2020). This enables the virus to be administered non-invasively and efficiently delivered to the tumor site, thereby avoiding systemic clearance and associated toxicities common with intravenous injection. Such an approach is relatively uncommon in the treatment of deep-seated solid tumors. Second, OVs have demonstrated notable efficacy in remodeling the tumor immune microenvironment in lung cancer (Dong et al., 2024; Volovat et al., 2024). This includes promoting infiltration of effector immune cells such as CD8^+^ T lymphocytes and enhancing responsiveness to combination therapies such as immune checkpoint inhibitors. Moreover, clinical trial data indicate a generally favorable safety profile for OVs, with the most common adverse events being mild to moderate, controllable influenza-like symptoms, which are substantially less severe than those typically observed with conventional chemotherapy (Lau et al., 2025). Nevertheless, the clinical translation of OV therapy for lung cancer continues to face substantial challenges. In contrast to melanoma, which has already seen regulatory approval of OV products such as T-VEC and completion of phase III trials, lung cancer lacks robust clinical evidence (Shalhout et al., 2023; Kalsi et al., 2024). Most studies remain in the early phases, and no OV products have been approved for lung cancer to date. Key clinical endpoints including objective response rate, progression-free survival (PFS), and overall survival have yet to be validated in large-scale clinical trials. Additionally, the current development pipeline is relatively underdeveloped, with a limited number of candidates under investigation and slow progress, resulting in restricted therapeutic benefits for patients. Several technical obstacles also hinder the application of OV therapy in lung cancer. Pulmonary tumors are often located deep within the lung or present as diffuse lesions, making intratumoral injection technically challenging. Procedures such as bronchoscopy or percutaneous puncture carry procedural risks and may not reach all tumor sites. Intravenous delivery is prone to immune clearance and sequestration by the liver and spleen, resulting in reduced delivery efficiency at the tumor site. Although inhalation delivery presents a promising alternative, it is limited by issues such as insufficient viral stability, difficulty penetrating the mucus barrier of the respiratory tract, and robust immune surveillance in the lungs (Kumbhar et al., 2022). Moreover, there is a lack of comprehensive data on viral biodistribution, replication efficiency, and long-term safety in pulmonary tissues. In comparison, melanoma presents a more favorable profile for OV therapy due to its superficial lesion location, ease of intratumoral injection, and inherently high immunogenicity (Wang et al., 2025). Furthermore, there is currently a lack of direct quantitative comparisons of immune responses following OV treatment between lung cancer and other tumor types. This limits the development of optimized therapeutic strategies. Finally, as a high-cost biologic therapy, the accessibility and cost-effectiveness of OV therapy in future clinical practice warrant careful consideration.

## 7 Discussion

OVs exhibit substantial promise in lung cancer therapy, leveraging their relative selectivity for tumor cell replication and lysis, activation of systemic antitumor immune responses, and reprogramming of the TME. Nonetheless, monotherapeutic OV strategies are constrained by inherent limitations, which combination therapies effectively mitigate—yielding synergistic enhancements in treatment efficacy. Although preclinical and early-phase clinical trials of combinatorial approaches have demonstrated encouraging results, the majority remain in developmental stages. Robust clinical validation of safety and efficacy, along with exploration of optimal combination regimens, is imperative for translational advancement. Several OVs have secured regulatory approval for cancer treatment, with numerous candidates in various stages of development. Future advancements are expected to yield an expanding repertoire of OVs for human disease therapy. However, market proliferation of OVs faces notable challenges, including administration-related constraints, absence of predictive biomarkers, and variability in clinical trial outcomes. A central focus for oncology research will be defining strategies to genetically engineer OVs and optimize combination therapies-balancing safety, efficacy, and minimization of adverse reactions. This endeavor is pivotal for driving breakthroughs in cancer therapeutics and advancing precision medicine paradigms.
